# Supplementary material for: Web-based design and analysis tools for CRISPR base editing
Source: BMC Bioinformatics. 2018 Dec 27;19:542. doi: 10.1186/s12859-018-2585-4 (PMC6307267; doi:10.1186/s12859-018-2585-4)

**Supplementary Figures**

**Web based design and analysis tools for CRISPR base editing**

Gue-Ho Hwang, Jeongbin Park, Kayeong Lim, Sunghyun Kim, Jihyeon Yu, Eunchong Yu, Sang-Tae Kim, Roland Eils, Jin-Soo Kim and Sangsu Bae*

**Contact:** sangsubae@hanyang.ac.kr

*Content:*

*Supplementary Figure 1.* The internal programs used in this study for implementation of BE-Designer and BE-Analyzer

*Supplementary Figure 2.* The workflow for classifying query sequences in BE-Analyzer

**Supplementary Figure 1.** The internal programs used in this study for implementation of BE-Designer and BE-Analyzer. (A) Schematic showing parameters for BE-Designer and BE-Analyzer. The summarized workflow and internal programs used in BE-Designer (B) and BE-Analyzer (C).


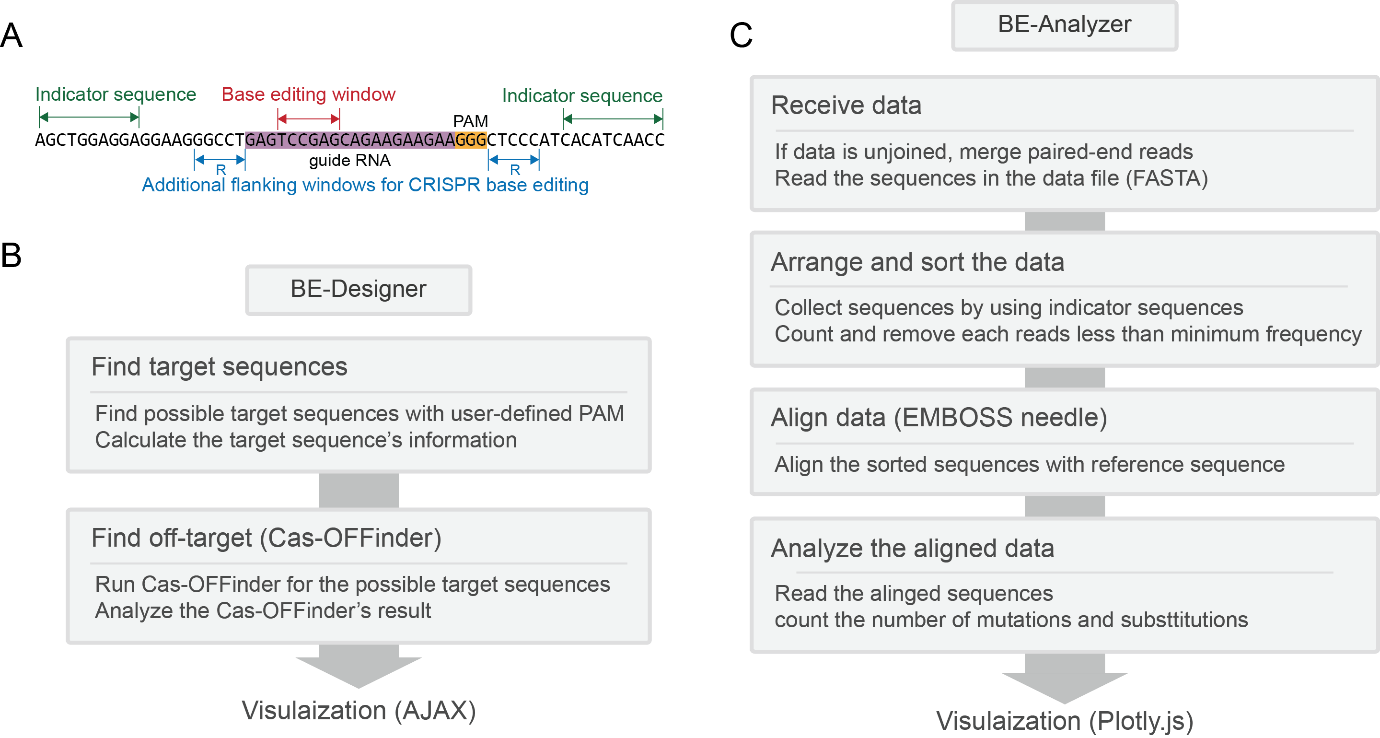


**Supplementary Figure 2.** The workflow for classifying query sequences in BE-Analyzer.


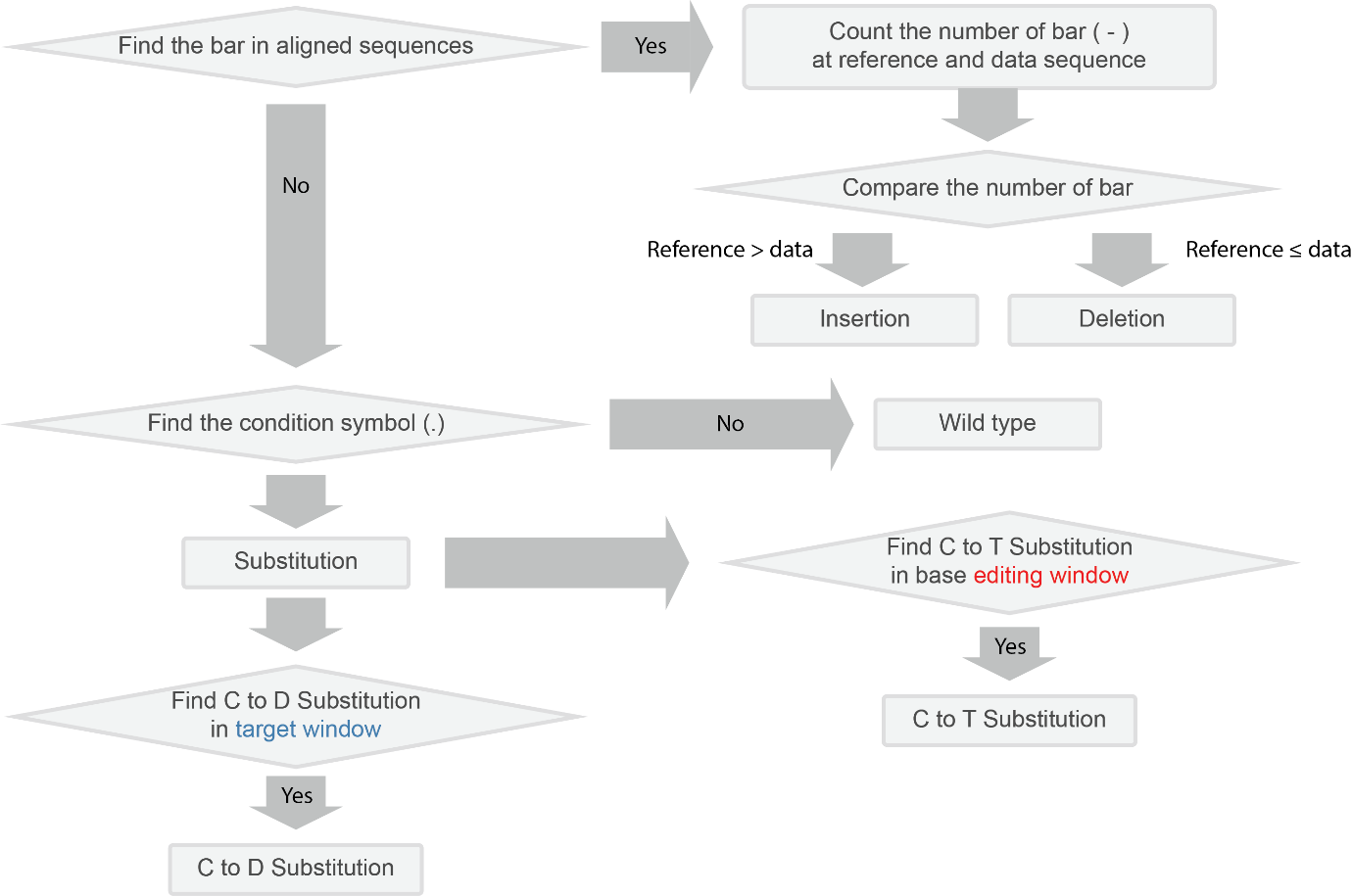

Supplement: Supplementary file 1 — Figure S1. The internal programs used in this study for implementation of BE-Designer and BE-Analyzer. Figure S2. The workflow for classifying query sequences in BE-Analyzer. (DOCX 338 kb) [file 12859_2018_2585_MOESM1_ESM.docx]
